# Decrease of *Staphylococcus aureus* Virulence by *Helcococcus kunzii* in a *Caenorhabditis elegans* Model

**DOI:** 10.3389/fcimb.2017.00077

**Published:** 2017-03-16

**Authors:** Christelle Ngba Essebe, Orane Visvikis, Marguerite Fines-Guyon, Anne Vergne, Vincent Cattoir, Alain Lecoustumier, Emmanuel Lemichez, Albert Sotto, Jean-Philippe Lavigne, Catherine Dunyach-Remy

**Affiliations:** ^1^Institut National de la Santé et de la Recherche Médicale, U1047, UFR de Médecine, Université de MontpellierNîmes, France; ^2^Team Microbial Toxins in Host Pathogen Interactions, Centre Méditerranéen de Médecine Moléculaire, C3M, Institut National de la Santé et de la Recherche Médicale, U1065Nice, France; ^3^Service de Microbiologie, CHU de CaenCaen, France; ^4^CNR de la Résistance aux Antibiotiques (Laboratoire Associé Entérocoques et Résistances Particulières chez les Bactéries à Gram Positif)Caen, France; ^5^Laboratoire de Biologie Médicale, CH CahorsCahors, France; ^6^Université de Caen NormandieCaen, France; ^7^Service de Maladies Infectieuses et Tropicales, CHU CarémeauNîmes, France; ^8^Service de Microbiologie, CHU CarémeauNîmes, France

**Keywords:** attenuation, *Caenorhabditis elegans*, co-infection, *Helcococcus kunzii*, *Staphylococcus aureus*, virulence

## Abstract

Social bacterial interactions are considered essential in numerous infectious diseases, particularly in wounds. Foot ulcers are a common complication in diabetic patients and these ulcers become frequently infected. This infection is usually polymicrobial promoting cell-to-cell communications. *Staphylococcus aureus* is the most prevalent pathogen isolated. Its association with *Helcococcus kunzii*, commensal Gram-positive cocci, is frequently described. The aim of this study was to assess the impact of co-infection on virulence of both *H. kunzii* and *S. aureus* strains in a *Caenorhabditis elegans* model. To study the host response, qRT-PCRs targeting host defense genes were performed. We observed that *H. kunzii* strains harbored a very low (LT50: 5.7 days ± 0.4) or an absence of virulence (LT50: 6.9 days ± 0.5). In contrast, *S. aureus* strains (LT50: 2.9 days ± 0.4) were significantly more virulent than all *H. kunzii* (*P* < 0.001). When *H. kunzii* and *S. aureus* strains were associated, *H. kunzii* significantly reduced the virulence of the *S. aureus* strain in nematodes (LT50 between 4.4 and 5.2 days; *P* < 0.001). To evaluate the impact of these strains on host response, transcriptomic analysis showed that the ingestion of *S. aureus* led to a strong induction of defense genes (*lys-5, sodh-1*, and *cyp-37B1*) while *H. kunzii* did not. No statistical difference of host response genes expression was observed when *C. elegans* were infected with either *S. aureus* alone or with *S. aureus* + *H. kunzii*. Moreover, two well-characterized virulence factors (*hla* and *agr*) present in *S. aureus* were down-regulated when *S. aureus* were co-infected with *H. kunzii*. This study showed that *H. kunzii* decreased the virulence of *S. aureus* without modifying directly the host defense response. Factor(s) produced by this bacterium modulating the staphylococci virulence must be investigated.

## Introduction

Diabetes mellitus is a worldwide public health problem representing the third cause of mortality and morbidity in the world (WHO, 2014). Foot ulcers are a common complication in diabetic patients. Indeed, 15–25% of diabetic patients will present foot ulcers during their life (Boulton et al., 2005). Infection of these ulcers is a frequent complication. It represents major causes of hospitalization, morbidity, and mortality. It is also one of the major causes of lower-limb amputation (Mayfield et al., 1998). Several studies have shown that diabetic foot ulcers (DFU) are polymicrobial (Dowd et al., 2008; Redel et al., 2013). However, *Staphylococcus aureus* represents the most frequent pathogen isolated in diabetic foot infections (DFI) (Gardner et al., 2013; Messad et al., 2013; Commons et al., 2015; Lesens et al., 2015; Dunyach-Remy et al., 2016; Hatipoglu et al., 2016). This Gram-positive coccus is a leading cause of a wide range of diseases from skin and soft tissue infections (e.g., impetigo, carbuncles) to life-threatening bacteraemia, toxic shock syndrome, endocarditis, and osteomyelitis (Lowy, 1998), for which it deploys an arsenal of virulence factors to destroy host immune cells and tissues (Tacconelli et al., 2006).

In DFI, *S. aureus* is associated with a great diverse community of bacterial species (e.g., enterobacteria, anaerobes, non fermentative Gram-negative bacilli, β-hemolytic streptococci, enterococci; Gardner et al., 2013). The transition between DFU and DFI is poorly understood. *S. aureus* can colonize and maintain the chronicity of the wounds but this state is transient. The knowledge of *S. aureus* pathogenicity reveals that this bacterium seems to be particularly adapted for soft tissue and bone infections. Indeed, the majority of infections remain localized to the feet notably in the toe bones (Dunyach-Remy et al., 2016). Social bacterial interactions are considered essential in numerous infectious diseases, including chronic wounds. These interactions have been described in all living entities (Brogden et al., 2005). For example, a model of synergistic effect between uropathogenic *Escherichia coli* and *Enterococcus faecalis* showed that *E. faecalis* increased the virulence of *E. coli* (Lavigne et al., 2008). Moreover, translocation of several enterobacteria isolates in the bloodstream results in higher mortality (Pittet et al., 1993). Interactions involving clonal or divergent strains of the same species have also been described (Parsek and Greenberg, 2005; Tourret et al., 2011). However, this type of documentation of bacterial interaction is scarce in DFU/DFI. If metagenomic technologies have determined that distinct communities of bacteria are present at different sites of the body, challenges remain in understanding the complex interplay of these different species in contributing to modify the bacterial virulence (Price et al., 2009; Gardner et al., 2013; Fernandez et al., 2015).

Recently the emergence of new tools (e.g., mass spectrometry, DNA pyrosequencing) in bacterial identification has highlighted the frequent association between *S. aureus* and *Helcococcus kunzii*, a catalase-negative, facultative anaerobic Gram-positive coccus in DFU (Haas et al., 1997; Chagla et al., 1998; Riegel and Lepargneur, 2003; Dowd et al., 2008; Lemaître et al., 2008; Park et al., 2014; Vergne et al., 2015). *H. kunzii* was first described as a non-pathogenic bacterium, likely member of the skin microbiome (Haas et al., 1997). This species is also known as an opportunistic pathogen that causes different types of infections (endocarditis, bacteraemia, meningitis, breast abscess, wound infections, prosthetic joint infections, osteomyelitis) in immunosuppressed patients (diabetic patient, drug fiend, alcoholic; Chagla et al., 1998; Lemaître et al., 2008; Park et al., 2014; Vergne et al., 2015). Nonetheless, the role of *H. kunzii* in the pathogenesis of cutaneous polymicrobial infections remains unknown. In this study, we sought to investigate the potential of virulence of *H. kunzii* strains isolated from DFU in a model of *S. aureus* induced infection of *Caenorhabditis elegans* (Irazoqui et al., 2010; Szabados et al., 2013; Visvikis et al., 2014; Messad et al., 2015). This model was previously used to study *S. aureus* virulence notably in strains isolated from DFU/DFI (Garsin et al., 2001; Sotto et al., 2012; Messad et al., 2015). Its pathogenicity in the worms was characterized by enterocyte effacement, intestinal epithelium destruction, and complete degradation of internal organs (Irazoqui et al., 2010) demonstrating the interest of this model in the study of bacterial-host interaction.

## Materials and methods

### Bacterial strains and growth conditions

The bacterial strains studied are listed in Table 1.

**Table 1:** **Fifty percentage Lethal Time of ***Caenorhabditis elegans*** infected with different ***S. aureus*** and two representative ***H. kunzii*** strains and evaluation of feeding behavior by measuring the pathogen avoidance**.

**Strain**	**Characteristics of the strain (References)**	**LT50 in days (IC95% inf-sup)**	**Occupancy test after 16 h (%)**	***P* OP50 vs. others**	***P* NSA739 vs. others**	***P* NSA1385 vs. others**	***P* Newman vs. others**
NSA1385	*S. aureus*, clinical, colonizing (Sotto et al., 2008)	4.7 (4.5–4.8)	98 ± 2	<0.001	<0.001	–	NS
NSA739	*S. aureus*, clinical, infecting (Sotto et al., 2008)	2.8 (2.4–3.0)	96 ± 4	<0.001	–	<0.001	<0.001
Newman	*S. aureus*, reference	4.3 (4.0–4.6)	95 ± 4	<0.001	<0.001	NS	–
H10	*H. kunzii*, clinical, colonizing (Vergne et al., 2015)	5.5 (4.6–6.4)	96 ± 4	<0.001	<0.001	<0.001	<0.001
H13	*H. kunzii*, clinical, colonizing (Vergne et al., 2015)	6.3 (5.8–6.8)	100 ± 0	NS	<0.001	<0.001	<0.001
OP50	*E. coli*, control strain	7.1 (6.6–7.7)	100 ± 0	–	<0.001	<0.001	<0.001

*The results are representative of at least five independent asssays for each group of strains. P, Pairwise comparison between LT50s using a log rank test; NS, not significant; LT50, 50% Lethal Time*.

A collection of 23 clinical isolates of *H. kunzii* collected from DFU in a multicentre study performed between February 2008 and August 2013 was used (Vergne et al., 2015). Moreover, to assess the co-infection between *H. kunzii* and *S. aureus*, in addition to the reference *S. aureus* strain Newman, two clinical *S. aureus* strains, both isolated and characterized in our hospital, were used: NSA1385 (a colonizing strain collected from uninfected ulcer) and NSA739 (an infecting strain collected from deep DFI; Sotto et al., 2008; Messad et al., 2015). *Escherichia coli* OP50 was used as control for nematodes. This bacterium is the standard feeding strain for Fer-15 nematodes. It harbors no known uropathogenic virulence factors.

The different bacteria were grown in Mueller-Hinton (MH) and Luria Bertani (LB) broth or agar at 37°C except *H. kunzii* strains which were grown on Columbia agar supplemented with 5% fresh sheep blood (bioMérieux, France) under a 5% CO_2_ atmosphere at 37°C during 48 h.

*S. aureus, H. kunzii* and *E. coli* grew identically alone or in association on the nematode growth medium (NGM) used to worms experiments at 37°C (Figure S1).

### Pulse field gel electrophoresis (PFGE)

PFGE analysis of genomic DNA fragments of the 23 clinical isolates of *H. kunzii* was carried out after digestion with the restriction endonuclease *Sma*I, as previously published for enterococci (Bourdon et al., 2011). The electrophoresis was performed using a CHEF-DRIII apparatus (BioRad, France) and PFGE patterns were interpreted according to well-established criteria (Tenover et al., 1995).

### Nematode killing assay

The nematode infection assay was carried out as previously described using the Fer-15 mutant line (a temperature sensitive fertility defect; Lavigne et al., 2008). Overnight cultures of the studied bacterial strains in the NGM were harvested, centrifuged and suspended in phosphate buffered saline solution (PBS) at a concentration of 10^5^ CFU/mL. Ten microliters of these bacterial suspensions were inoculated on NGM agar plates. A ratio 1:1 (10^5^/10^5^) was prepared during co-infection assays. To validate this ratio, we evaluated the CFU of both bacteria prior to the seeding onto agar to confirm that *S. aureus* and *H. kunzii* were present in an equal amount. The plates were incubated at 37°C for 8−10 h. Around 30 L4 stage nematodes per plate were thus seeded and then incubated at 25°C. An independent reader (blind to the culture) scored each day the number of live nematode under a stereomicroscope (Leica, France).

### Effect of sequential infection of *C. elegans*

*C. elegans* were infected with two representative *H. kunzii* strains (H10 with no virulence and H13 with a low virulence). After 12 h, 30 nematodes were transferred to NGM medium containing the different *S. aureus* strains. In the same way, to evaluate the bacterial persistence, nematodes were coinfected with *H. kunzii* and *S. aureus*. After 12 h, 30 nematodes were transferred to NGM medium containing OP50 strain. The conditions of nematodes preparation were strictly similar to previous assays described before. Final analysis established the Lethal time 50% (LT50), which corresponds to time (in days) required to kill 50% of the worms.

Three replicates repeated five times were performed for each studied strain.

### Feeding behavior experiments

Firstly, all the studied bacterial strains were grown in LB broth media (with or without anaerobes conditions) at 25°C during 16 h. The cultures were then spotted on NGM plates. Around 30 L4 stage nematodes were deposed in the center of the bacteria lawn. To establish the occupancy assays, the number of nematodes inside or outside each lawn was counted after overnight incubation as previously published (Lavigne et al., 2013). The results were presented in percent occupancy (number of worms in the bacterial lawn on the total number of *C. elegans*). The experiments were performed in triplicate.

Secondly, we determined the number of bacteria within the nematode gut (Garsin et al., 2001; Lavigne et al., 2008). Briefly, nematodes were picked at 72 h, and the surface bacteria were removed by washing the nematodes twice in M9 medium containing 25 μg/ml gentamicin. The *C. elegans* were then mechanically disrupted in M9 medium containing 1% Triton X-100. Finally, after serial dilutions, 100 μl of the mixture were plated on LB agar medium and on Columbia agar supplemented with 5% fresh sheep blood (when the co-infection experiments were performed). The colonies [aspect and haemolytic activity (α haemolytic for *H. kunzii* and β haemolytic for *S. aureus*)] were counted after 24 h and the identification of each species was confirmed by MALDI-TOF (Vitek MS, BioMérieux). Three replicate assays were performed for each strain.

### Effect of *H. kunzii* on *S. aureus* virulence genes expression

Analysis of the mRNA levels of *spa, hla*, and *agr* was performed following the method previously published (Doumith et al., 2009; Kriegeskorte et al., 2014). These 3 genes are essential in the *S. aureus* pathogenicity notably in worms model (Sifri et al., 2003, 2006). *S. aureus* isolates and *H. kunzii* H13 were grown alone or in association in MH broth to an OD_600_ of ~0.7. The total RNA extraction was performed using the RNeasy Mini kit (Qiagen, France) during exponential stages. Purity and concentration were determined by the NanoDrop 2000 spectrophotometer (Fisher Scientific, USA). The iScript^*TM*^ Select cDNA Synthesis Kit (Biorad, Hurcules, CA) was used to the synthesis of cDNA from 1 μg of total RNA for each sample. Real-time PCR were done in a LightCycler® 480 (Roche, France) using the LightCycler FastStart DNA Master^PLUS^ SYBRGreen I kit, 100 ng of cDNA and 10 pmol of target primers (Table 4). Amplifications were analyzed in triplicate from three different RNA preparations. Cycle threshold (*Ct*) values of the different target genes were compared with the *Ct*-values of the house-keeping gene (*gyrB*) (Sihto et al., 2014). The Newman strain was used as control. The normalized relative expressions of the studied genes were obtained for each strain following the equation: 2^−ΔΔ*Ct*^ (ΔΔCt = (Ct_gene_–Ct_*gyrB*_)_studied strain_—(Ct_gene_–Ct_*gyrB*_)_control strain_) (Livak and Schmittgen, 2001). Results obtained for each gene were log-transformed to obtain a fold change difference between strains.

### Evaluation of host response by quantitative real time-PCR

For selected genes involved in *C. elegans* response against infection [*hlh-30, lys-5, lgg-1, clec-7* (Visvikis et al., 2014), *cyp-37B1* (Irazoqui et al., 2010), *sodh-1* (JebaMercy and Balamurugan, 2012)], transcript level analysis was performed by qRT-PCR following the same protocol described before. *C. elegans* were infected between 12 h with studied bacterial strains. The nematodes were then washed twice in water. Total RNA from animals was extracted by using TRIzol® RNA Isolation Reagents (ThermoFisher, France). The target primers used were presented in Table 4. The 2^−ΔΔ*CT*^ method was used to analyze transcriptional changes in target genes using *snb-1* as the housekeeping control gene (Livak and Schmittgen, 2001). Data analysis was performed with the Pfaffl method (Pfaffl, 2001). Error bars indicate the standard deviation (SD) of three independent experiments.

### Statistical analysis

Statistics and graphs were performed using GraphPad Prism 6.0 software.

For the nematode killing experiments, a log-rank (Mantel-cox) test was used to evaluate differences in survival rates between the different strains.

Log-transformed data were used for RT-PCR. The effects of bacterial infections on expression of selected genes involved in *S. aureus* virulence and in host response were performed using one-way ANOVA followed by Dunnett's multiple comparisons test. A statistically significant difference was retained for *P* < 0.01.

## Results

### Virulence of *H. kunzii* and *S. aureus* strains

The virulence of a clinical panel of 23 *H. kunzii* isolates was evaluated in a nematode model. The genetic comparison of the 23 strains showed that the isolates were not clonally related (Figure S2) eliminating a clonal impact of the virulence behavior. Out of 23, 17 (74%) *H. kunzii* strains were non-virulent with a behavior similar to the non-pathogenic *E. coli* OP50 (LT50s: 6.1 days ± 0.4 vs. 7.1 days ± 0.5, respectively), a laboratory reference strain used to feed nematodes [*P*-value, non significant (NS)]. The other six *H*. *kunzii* strains (H7, H10, H17a, H20, H21, H22b) were significantly more virulent than OP50 (*P* < 0.001; Table 1, Table S1).

To compare the virulence between *H. kunzii* and *S. aureus*, we used well-characterized *S. aureus* strains: the *S. aureus* strain NSA739 (collected from DFI and harboring a high virulence potential), the *S. aureus* strain NSA1385 (collected from DFU and harboring a low virulence potential) and the *S. aureus* reference strain Newman (Sotto et al., 2008; Messad et al., 2015). We observed that the panel of *H. kunzii* presented significantly lower virulence than NSA739 (LT50: 2.8 days ± 0.4), NSA1385 (LT50: 4.7 ± 0.2) and Newman (LT50: 4.3 ± 0.3; *P* < 0.001), respectively. The difference of virulence between the infecting and the colonizing strains of *S. aureus* was previously demonstrated (Table 1, Table S1; Messad et al., 2015). These results confirmed that *H. kunzii* strains are low- or non-virulent bacteria.

### Decrease of *S. aureus* virulence by *H. kunzii*

**Figure 1:**
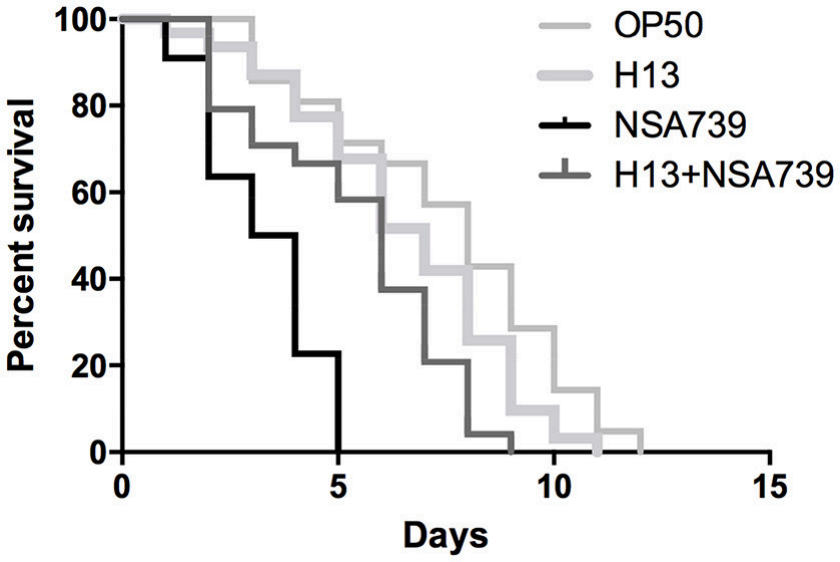
*****In vivo*** kinetics of killing of ***C. elegans*** infected by ***S. aureus*** NSA739, ***H. kunzii*** H13, and co-infected by the two strains**. OP50 represents the survival curve for worms fed on non-pathogenic *E. coli*. In all cases, worms were grown on NGM plates at 25°C and ≈ 30 Fer-15 were used in each test. The curves are representative of at least three independent trials for each group of strains.

When the different *H. kunzii* strains and NSA739 were used to co-infect *C. elegans*, an important attenuation of the *S. aureus* virulence was observed independently of *H. kunzii* virulence or non-virulence potential (Table 2 Table S2, Figure 1). The LT50 obtained with the strains coinfection varied between 4.1 and 5.7 days. They were significantly longer than the LT50 detected with *S. aureus* strain alone (LT50: 2.8 days; *P* < 0.001).

**Table 2:** **Fifty percentage Lethal Time of ***Caenorhabditis elegans*** co-infected with a virulent ***S. aureus*** strain (NSA739) and two representative ***H. kunzii*** strains and evaluation of feeding behavior by measuring the pathogen avoidance**.

**Strain**	**LT50 in days (IC95% inf-sup)**	**Occupancy test after 16 h (%)**	***P* OP50 vs. others**	***P* NSA739 vs. others**	***P* H10 vs. others**	***P* H13 vs. others**
NSA739	2.8 (2.4–3.0)	96 ± 4	<0.001	–	<0.001	<0.001
H10	5.5 (4.6–6.4)	96 ± 4	<0.001	<0.001	–	NS
H13	6.2 (5.8–6.6)	100 ± 0	NS	<0.001	NS	–
H10+ NSA739	4.1 (4.0–4.3)	92 ± 5	<0.001	<0.001	<0.001	<0.001
H13+ NSA739	5.7 (5.3–5.9)	100 ± 0	<0.001	<0.001	<0.001	NS
H10> + OP50 ^ψ^	6.6 (6.2–6.8)	100 ± 0	NS	<0.001	<0.001	NS
H13> + OP50^ψ^	6.2 (5.8–6.6)	97 ± 3	NS	<0.001	NS	NS
NSA739> + OP50^ψ^	2.8 (2.4–3.0)	96 ± 4	<0.001	NS	<0.001	<0.001
NSA1385> +OP50^ψ^	4.4 (4.0–5.1)	100 ± 0	<0.001	<0.001	<0.001	<0.001
Newman> +OP50^ψ^	4.0 (3.5–4.3)	94 ± 4	<0.001	<0.001	<0.001	<0.001
H10> +NSA739^*^	2.5 (2.4–2.7)	100 ± 0	<0.001	NS	<0.001	<0.001
H13> +NSA739^*^	4.1 (3.7–4.4)	100 ± 0	<0.001	<0.001	<0.001	<0.001
OP50	7.1 (6.6–7.7)	100 ± 0	–	<0.001	<0.001	<0.001

The results are representative of at least four independent assays for each group of strains. P, Pairwise comparison between LT50s using a log rank test; NS, not significant; LT50, 50% Lethal Time. Infection of nematodes with H10 or H13 followed by transfer on

ψ OP50 or

* *S. aureus 12 h after*.

To confirm the role of *H. kunzii* in the diminution of *S. aureus* virulence, we tested the effect of *H. kunzii* on two others *S. aureus* strains: NSA1385 (colonizing strain) and reference strain Newman and on the *E. coli* OP50. If the majority of *H. kunzii* strains had statistically no impact on *S. aureus* virulence, 8 strains (H4, H6, H8, H13, H16, H17b, H22b, H23) reduced significantly the virulence of NSA1385 (LT50: 5.6–6.3 days; *P* < 0.001; Table 3, Table S3). On the other hand, we observed that 6 *H. kunzii* strains (H9, H13, H18, H22a, H22b, H23) reduced the virulence of the reference strain Newman (LT50: 5.8–7.1 days; *P* < 0.001; Table 3). Interestingly no difference could be noted when worms were fed with *H. kunzii* + OP50 (Table 3), suggesting a specific effect between *S. aureus* and *H. kunzii*.

**Table 3:** **Fifty percentage Lethal Time of ***Caenorhabditis elegans*** co-infected with ***S. aureus*** strains and two representative ***H. kunzii*** strains and evaluation of feeding behavior by measuring the pathogen avoidance**.

**Strain**	**LT50 in days (IC95% inf-sup)**	**Occupancy test after 16h (%)**	***P* OP50 vs. others**	***P* NSA1385 vs. others**	***P* Newman vs. others**	***P* H10 vs. others**	***P*H13 vs. others**
NSA1385	4.7 (4.5–4.8)	98 ± 2	<0.001	–	NS	NS	<0.001
Newman	4.3 (4.0–4.6)	95 ± 4	<0.001	NS	–	<0.001	<0.001
H10	5.5 (4.6–6.4)	96 ± 4	<0.001	NS	<0.001	–	NS
H13	6.2 (5.8–6.6)	100 ± 0	NS	<0.001	<0.001	NS	–
H10 + NSA1385	4.0 (3.9–4.2)	100 ± 0	<0.001	NS	–	<0.001	<0.001
H10 + Newman	3.6 (3.5–3.7)	90 ± 5	<0.001	<0.001	NS	<0.001	<0.001
H13 + NSA1385	5.8 (5.7–5.9)	100 ± 0	<0.001	<0.001	–	NS	NS
H13 + Newman	6.3 (6.2–6.4)	90 ± 5	NS	<0.001	<0.001	NS	NS
H10 + OP50	5.6 (5.1–6.0)	100 ± 0	<0.001	NS	<0.001	NS	NS
H13 + OP50	6.4 (6.0–6.6)	100 ± 0	<0.001	<0.001	<0.001	NS	NS
H10> + NSA1385^*^	4.9 (4.7–5.1)	100 ± 0	<0.001	NS	–	NS	<0.001
H10> + Newman^*^	4.6 (4.3–4.8)	95 ± 4	<0.001	NS	NS	NS	<0.001
H13> + NSA1385 ^*^	5.2 (5.0–5.3)	100 ± 0	<0.001	NS	–	NS	<0.001
H13> + Newman^*^	5.5 (5.2–5.7)	92 ± 3	<0.001	NS	<0.001	NS	NS

The results are representative of at least four independent assays for each group of strains. P, Pairwise comparison between LT50s using a log rank test; NS, not significant; LT50, 50% Lethal Time. Infection of nematodes with H10 or H13 followed by transfer on

* *S. aureus 12 h after*.

**Table 4:** **Primers used in the study**.

**Primer use and target function**	**Target region**	**Primer name**	**Oligonucleotide sequence**	**Tm (°C)**	**References**
**HOST qRT-PCR**					
Transcriptional factor for host defense	*hlh-30*	hlh-30 F	5′-CGGGCTGGCTCAGGACACTC-3′	65.5	Visvikis et al., 2014
		hlh-30 R	5′-GGCGCCGAACTTGAGACGAC-3′	63.5	
Antimicrobial function	*lys-5*	lys-5 F	5′-CGGGCTGGCTCAGGACACTC-3′	54.7	Visvikis et al., 2014
		lys-5 R	5′-GGCGCCGAACTTGAGACGAC-3′	53.2	
	*clec-7*	clec-7 F	5′-TTTATGGGACGATTCGACGG-3′	57.3	Visvikis et al., 2014
		clec-7 R	5′-GTCAATGCACCTTGTACGGA-3′	57.3	
Cytoprotection	*cyp-37B1*	cyp-37B1 F	5′-GAATGTATCCGTCAGTGCCA-3′	57.3	Irazoqui et al., 2010
		cyp-37B1 R	5′-TCGGACTCCTTTTGGGAAGA-3′	57.3	
Detoxification	*sodh-1*	sodh-1 F	5′-CTGGATGGCAACTTGGAGACAAAGC-3′	64.6	Irazoqui et al., 2008
		sodh-1 R	5′-GGTGGCAGAGTGGCTCGTGG-3′	65.5	
Autophagy	*lgg-1*	lgg-1 F	5′-ACCATGACCACAATGGGACAACTC-3′	62.7	Visvikis et al., 2014
		lgg-1 R	5′-ACACTTTCGTCACTGTAGGCGATG-3′	62.7	
***S. aureus*** **qRT-PCR**					
α hemolysin	*hla*	Hla-F	5′-TCCAGTGCAATTGGTAGTCA-3′	55.3	Otto et al., 2013
		Hla-R	5′-GGCTCTATGAAAGCAGCAGA-3′	57.3	
Protein A	*spa*	Spa-F	5′-TATGCCTAACTTAAATGCTG-3′	51.1	Otto et al., 2013
		Spa-R	5′-TTGGAGCTTGAGAGTCATTA-3′	53.2	
Accessory gene regulator	*agrA*	F_agrA_34	5′-CAAAGAGAAAACATGGTTACCATTATTAA-3′	58.2	Garzoni et al., 2007
		R_agrA_135	5′- CTCAAGCACCTCATAAGGATTATCAG-3′	61.6	

All these findings strongly suggest the important role of particular *H. kunzii* strains in the attenuation of *S. aureus* virulence isolated from wounds notably for highly virulent *S. aureus* strains.

### Effect on feeding behavior

**Figure 2:**
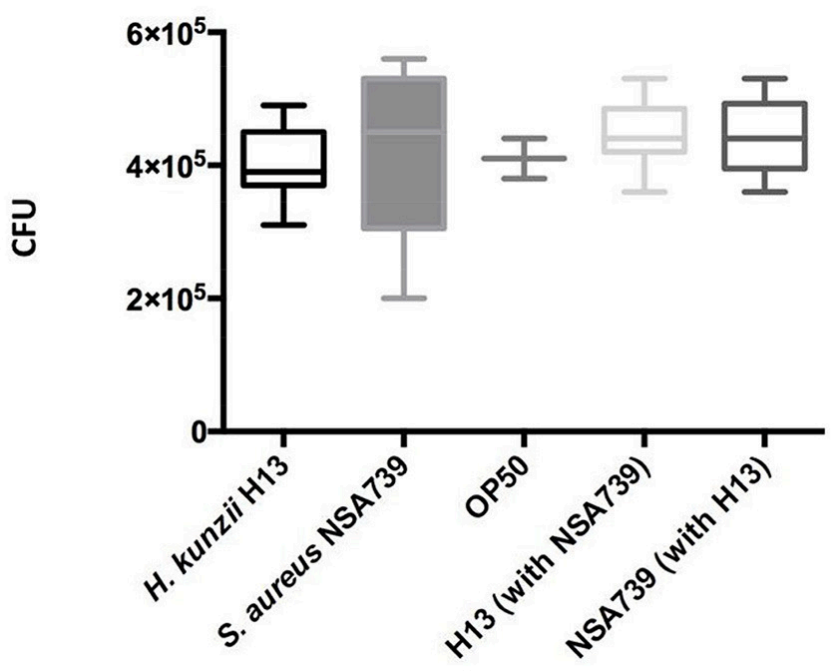
**Evaluation of feeding behavior by measuring bacterial content of ***C. elegans*****. Each graph represents the median of CFU count per worm found in each species alone (the *H. kunzii* H13, the *S. aureus* NSA739, and the *E. coli* OP50) or in co-infection (*H. kunzii* H13 + *S. aureus* NSA739). Three replicates were performed for each strain alone or in association. Differences in CFU rates were tested by a Pearson normality test. These results are representative of the data obtained for all the strains evaluated in this study.

To exclude the possibility that the observed results in worms was due to a modification of their feeding behavior, an occupancy test was performed. None of the bacterial strains tested alone or in association presented strong avoidance behavior. No significant difference was noted in the fraction of nematodes on the bacterial lawn between the different associations studied (Tables 1–3, Tables S1–S3). We also measured the bacterial load in the intestine of nematodes at 72 h post infection (Lavigne et al., 2013). We found that all bacteria tested alone or in association can colonize and survive in the *C. elegans* intestine (Figure 2). The number of the *H. kunzii* and *S. aureus* CFU was around 4 × 10^5^ bacteria per nematode (*IC*95% = 2.8–7.9 × 10^5^) within the nematode intestine for each combination without statistical difference (*P*-value, NS).

These results confirm the low virulence of *H. kunzii* strains and suggest that the impact on the modulation of *S. aureus* virulence observed in *C. elegans* was not due to a modification of nematodes' feeding behavior, nor to a reduction of *S. aureus* infection rate or a hypothetical cytotoxicity effect of *H. kunzii* on *C. elegans*.

### Transcriptional host response during co-infection between *H. kunzii* and *S. aureus*

To estimate the host response during co-infection between *H. kunzii* and *S. aureus*, we carried out qRT-PCRs on six representative host defense genes of nematodes after infection by *H. kunzii* and *S. aureus* alone or in co-infection: *hlh-30* (the key transcriptional factor-encoded gene for *S. aureus* host defense), *lys-5* and *clec-7* (antimicrobial-encoded genes), *cyp-37B1* and *sodh-1* (cytoprotective and detoxification-encoded genes) and *lgg-1* (autophagy-encoded gene). Of the different co-infection combinations we choose to study one *H. kunzii* isolate non-virulent in nematode model and reducing the virulence of all *S. aureus* strains (H13) and one with low virulence in nematode model and that had effect exclusively on NSA739 virulence (H10).

**Figure 3:**
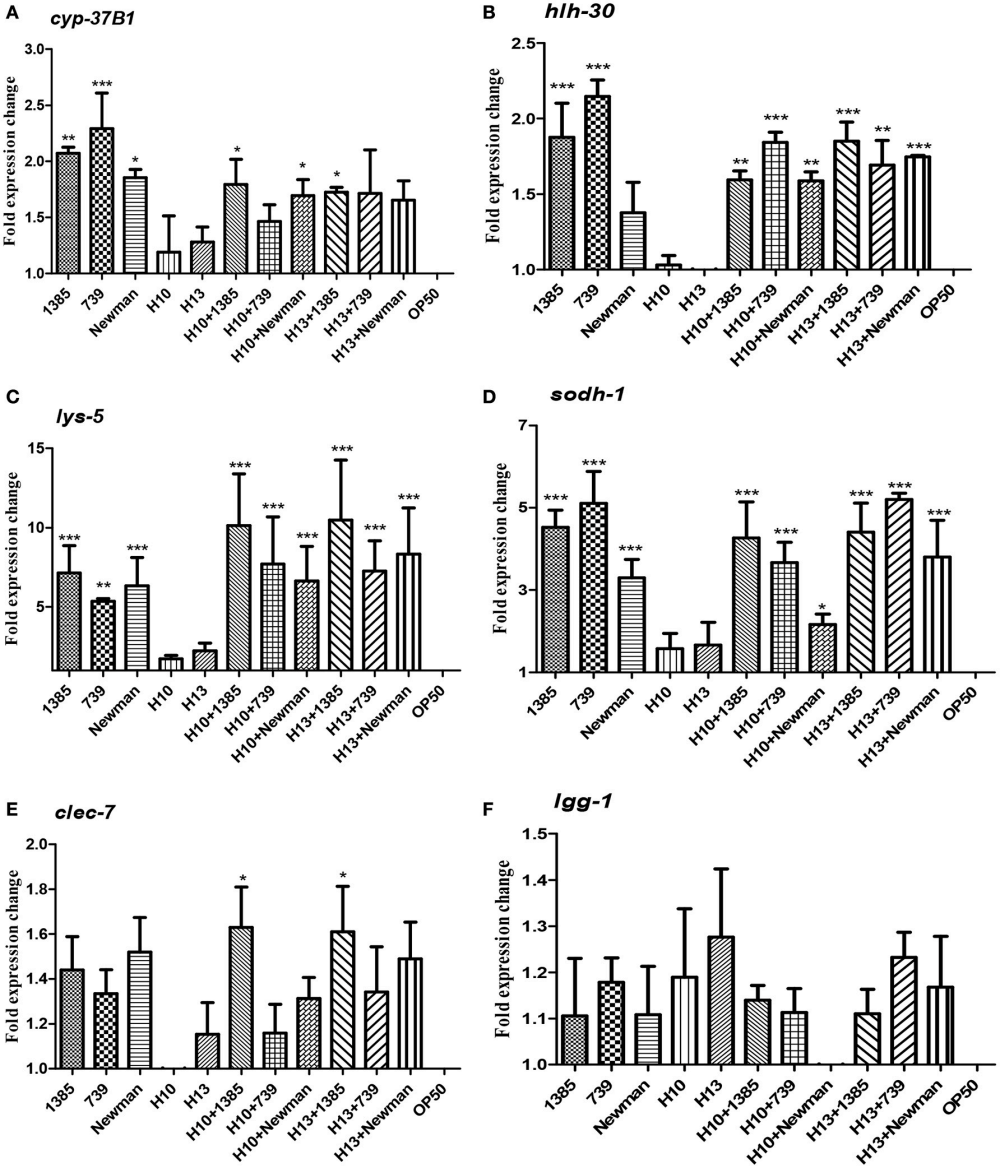
*****C. elegans*** L4 host response to ***S. aureus*** and ***H. kunzii*** infections alone or in association at 12 h post infection**. The figure represents the expression of six main genes involved in *C. elegans* immune response: *cyp-37B1* **(A)**, *hlh-30* **(B)**, *lys-5* **(C)**, *sodh-1* **(D)**, *clec-7* **(E)**, *lgg-1* **(F)**. Data are expressed as the mean ± sd of three biological replicates of qRT-PCR results. All *Ct*-values are normalized against the housekeeping gene *snb-1*. Data analysis was performed with the Pfaffl method (Pfaffl, 2001). The *P*-value represents the comparison between genes expression found in *H. kunzii* strains alone and the other combination (*S. aureus* alone, co-infection *H. kunzii*+*S. aureus*) ^*^P < 0.05; ^**^P < 0.01; ^***^P < 0.001.

We found that nematodes fed with the two *H. kunzii* strains did not show significant differences of expression of host defense genes compared to nematodes fed with the non-pathogenic strain OP50. This result confirms that *H. kunzii* strains do not stimulate the *C. elegans* immune response (Figure 3). On the other hand, when nematodes were fed with the three *S. aureus* strains, they significantly overexpressed *hlh-30, lys-5, sodh-1*, and *cyp-37B1* compared to nematodes fed with OP50 (*P* < 0.01). Only the autophagy gene *lgg-1* had no modification of expression whatever the strain and the condition tested (*P*-value, NS). Interestingly, no significant difference in the expression of host response genes could be observed between each *S. aureus* strains tested (colonizing or infecting; Figure 3). These results confirm a nematode host response when *C. elegans* were infected with *S. aureus*.

Finally, when we co-fed nematodes with *H. kunzii* and *S. aureus* strains, we observed that the majority of host defense genes (*hlh-30, lys-5, cyp-37B1*, and *sodh-1*) were overexpressed compared to OP50 or *H. kunzii* alone (*P* < 0.01). Gene expression levels were equivalent to those observed with nematodes fed with *S. aureus* alone (*P*-value, NS; Figure 3). Surprisingly the *clec-7* gene has only significant variation of expression when *C. elegans* were fed with the colonizing *S. aureus* strain NSA1385 (*P* < 0.05).

These results suggest that the co-infection with *H. kunzii* and *S. aureus* induced an overexpression of some host defense genes. However, the variation of expression of *clec-7* gene during the coinfection of *H. kunzii* and the virulent/non-virulent *S. aureus* strains could suggest some modulations of host defense. So, during the coinfection, the attenuation of *S. aureus* virulence in presence of *H. kunzii* seems to not be due to its capacity to trigger *C. elegans* host response that would help the fight against *S. aureus* infection. *H. kunzii* seems to directly act in the modulation of *S. aureus* virulence and to have no major role on the modulation of host immune defense.

### Effect of *H. kunzii* on *S. aureus* virulence genes expression

**Figure 4:**
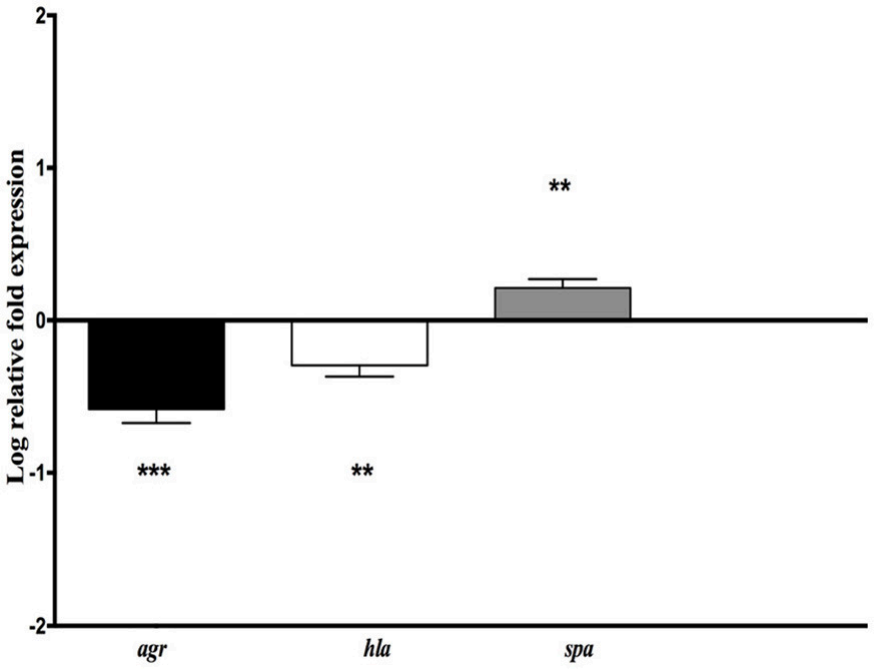
**Relative mRNA expression level of genes implicated in virulence (***hla***, ***spa***) and virulence regulation (***agr***) for ***S. aureus*** NSA739 co-infected with ***H. kunzii*** H13**. The log-transformed averages of relative fold change of *S. aureus*+*H. kunzii* co-infection compared to *S. aureus* alone are presented. The error bars represent the standard deviation from three different RNA preparations. Significant differences from *S. aureus* co-infected with *H. kunzii* using Dunnett's test are indicated by ^**^(*p* < 0.01) and ^***^ (*p* < 0.001).

To look into the possibility of direct attenuation of *S. aureus* virulence by *H. kunzii*, the expression levels of two representative virulence genes (*hla* and *spa*) and the main regulatory gene *agr* (that influences the expression of numerous *S. aureus* virulence genes) were measured for the different *S. aureus* strains associated with the *H. kunzii* isolate H13 and compared to those of *S. aureus* alone (Figure 4).

We observed that *hla* gene which encodes for the α-hemolysin (representing one of the most important virulence factors) was significantly derepressed in *S. aureus* NSA739 associated with *H. kunzii* H13 [Median −0.277; 95%CI (−0.41/−0.18); *p* < 0.01]. In the same way, *agr* was also significantly down regulated [Median −0.582; 95%CI (−0.36/−0.81); *p* < 0.001]. *spa* gene which encodes the protein A (representing one of the most important colonizing factor) showed a significant overexpression in *S. aureus* NSA739 associated with *H. kunzii* H13 [Median 0.197; 95%CI (0.15–0.27); *p* < 0.001]. The same results were noted for the two other *S. aureus* studied.

This data suggested that *H. kunzii* attenuated directly the virulence of *S. aureus* by a deregulation of virulence genes and the global regulator of this virulence.

### Effect of sequential infections of *C. elegans*

To get a better understanding of the role of *H. kunzii* on *S. aureus* virulence, we evaluated the effect of sequential infections on nematodes. Firstly, we infected *C. elegans* with the different bacteria alone and in association during 12 h followed by a transfer of nematodes on OP50 strain. The results showed that *S. aureus* is clearly more virulent compared to *H. kunzii* (*P* < 0.001) and this virulence was not due to a constant reinfection since all the results were comparable to those obtained in the first experiments (Tables 2, 3).

Secondly, we sequentially inoculated the different associated bacteria. We infected *C. elegans* with the *H. kunzii* strains alone during 12 h followed by a transfer of nematodes on the different *S. aureus* strains tested. We observed that the LT50s for this protocol were significantly reduced (LT50: 2.5–4.1 vs. 4.1–5.7 days, respectively; *P* < 0.001). However, this impact was not clearly equivalent for the different combinations tested. Indeed, for the *H. kunzii* strain (H10) with different impact on the *S. aureus* virulence, the LT50 was similar to LT50 obtained for nematodes infected by NSA739 alone (LT50: 2.5 vs. 2.8 days, respectively; *P*-value, NS). For the *H. kunzii* strain (H13) with an impact on the virulence of all the *S. aureus* studied, LT50 remained significantly reduced compared to nematodes infected with NSA739 alone (LT50: 4.1 vs. 2.8 days, respectively; *P* < 0.001; Table 2). Thus, the direct association of *H. kunzii* and *S. aureus* has an impact on the attenuation of *S. aureus* virulence. This effect is significantly reduced or aborted when the infection is sequential suggesting the necessity to simultaneously co-infect with both *H. kunzii* and *S. aureus* to attenuate the virulence of *S. aureus*. *H. kunzii* seems to act directly on *S. aureus* reducing its virulence and thus the host response (showed by the reduction of *cyp-37B1* and *clec-7* expression previously).

## Discussion

Social interactions involving parasites, protozoa and prokaryotes have been frequently described (Tourret et al., 2011). Microbial co-occurrence networks indicate that bacterial species co-infect the same site of the human body and form microbial communities (Fernandez et al., 2015). However, documentations concerning interactions between non-virulent and pathogenic microorganisms are scarce particularly in DFU. In this article we show for the first time evidence of the modulation of *S. aureus* virulence when associated with a commensal bacterium, *H. kunzii*, frequently found associated in chronic wounds of the lower limbs (Vergne et al., 2015).

Some studies described the interactions between *S. aureus* and other pathogens (Hoffman et al., 2006; Baldan et al., 2014; Nair et al., 2014; Zago et al., 2015; Frydenlund Michelsen et al., 2016). These interactions vary between the microorganisms: cooperation with *E. faecalis* and *Candida albicans* (Engelmann et al., 2011; Nair et al., 2014; Zago et al., 2015), competition with *Lactobacillus* sp. (Ortiz et al., 2014), and *Streptococcus pneumoniae* (Margolis et al., 2010). *S. aureus* can also have both interactions (competition and cooperation) with the same pathogen depending of the disease and the conditions such as *Pseudomonas aeruginosa* (Hoffman et al., 2006; Baldan et al., 2014; Serra et al., 2015; Frydenlund Michelsen et al., 2016). If *P. aeruginosa* seems to never coaggregate with *S. aureus* in chronic wound ulcers (Fazli et al., 2009), these bacteria could share some siderophores to favor the growth of each other (Harrison et al., 2008). Moreover, our team has recently demonstrated the coexistence of two *S. aureus* population on DFU notably one with a very low virulence potential (Messad et al., 2015). In this context, the study of the effect of *H. kunzii* is of particular interest. Although we confirmed that this microorganism has a low virulence potential in the nematode model (74% tested strains were non-virulent and 26% harbored a low-virulence profile), some studies have described that *H. kunzii* can also be an opportunistic pathogen (Lemaître et al., 2008), notably in chronic wounds (Riegel and Lepargneur, 2003; Moore et al., 2010; Stanger et al., 2015; Vergne et al., 2015). Its frequent association with *S. aureus* on DFU reinforced the need of a better understanding of the cooperation mechanisms between the two bacteria. Here, we observed that all the *H. kunzii* isolates associated with a virulent *S. aureus* strains (NSA739) clearly increased the lifespan of the *C. elegans* (LT50s: 4.1–5.7 vs. 2.8 days, *P* < 0.001). This effect was confirmed when the nematodes were infected with *H. kunzii* and two other less virulent *S. aureus* strains (the reference strain Newman and a DFU colonizing strain NSA1385). Moreover, this effect seems to be specific to *S. aureus* while no effect could be observed when *H. kunzii* were associated with *E. coli* OP50. To explain these results, two hypotheses could be made: (i) *H. kunzii* modulated the immune response of *C. elegans* and help them to be more resistant or tolerant to *S. aureus*, (ii) *H. kunzii* modulated directly the *S. aureus* virulence.

Previous experiments showed that primary infection with *S. aureus* can increase vulnerability of *C. elegans* and modify its tolerance to an opportunistic pathogen *Proteus mirabilis* (JebaMercy and Balamurugan, 2012). The sequential infections of nematodes provide the evidence that *H. kunzii* does not affect the tolerance of *C. elegans* to *S. aureus*. The fact that nematodes tolerate more *S. aureus* when they are mixed with *H. kunzii* could be due to a direct interaction between the two strains. *H. kunzii* may have a direct effect on *S. aureus* by interfering with the expression of *S. aureus* virulence genes. To confirm this hypothesis, we analyzed the expression of main genes involved in nematode defense response after infection with *S. aureus, H. kunzii*, and both (Irazoqui et al., 2010; Visvikis et al., 2014). If *H. kunzii* strains did not modify these genes expression, *S. aureus* strains had a clear effect on the expression of *C. elegans* host defense genes particularly *hlh-30, cyp37, lys-5*, and *sodh-1*, whatever the virulence of the strain. This observation is consistent with two studies showing that after 8 h of infection with *S. aureus, C. elegans* modified the production of defense genes (*clec-71, sodh-11, cyp-37B1, lys-5*) that have xenobiotic detoxification potential or antimicrobial activities, and then protect host by participating to host response (Irazoqui et al., 2010; Visvikis et al., 2014). Also, our results show that *H. kunzii* does not modulate the immune response of *C. elegans* and the effect observed was due to a direct interaction between *H. kunzii* and *S. aureus* virulence. The downregulation of *hla* and *agr* expression in *S. aureus* co-cultured with *H. kunzii* sustained this hypothesis. In presence of *H. kunzii, S. aureus* could be in a “colonizing” behavior (as suggested by the overexpression of *spa* gene). Taken together, our work also confirms that *C. elegans* are not just a simple model to study pathogens' virulence. It is an entire organism that can establish immune mechanism to fight against infection and depending to the pathogen agent, can stimulate host defense genes (Irazoqui et al., 2010; Engelmann et al., 2011; Visvikis et al., 2014). Nematodes use some metabolic pathway of defense and express some genes that share similarities and/or homologies with those expressed during vertebrate and human infection (Irazoqui et al., 2010). Even if a low number of host and bacterial genes have been evaluated, the 6 selected *C. elegans* host genes and the 3 selected *S. aureus* virulence genes have been previously demonstrated as essential in the study of host-pathogen interaction (Sifri et al., 2003, 2006; Irazoqui et al., 2010; Visvikis et al., 2014). Further investigations need to be carried out in order to define by which mechanism(s) *Helcococcus* may alter *S. aureus* virulence.

To the best of our knowledge this is the first description of a virulence-modulating bacterial interaction between a non-virulent bacterial species and a naturally occurring pathogenic strain. This virulence attenuation was independent to host defense mechanisms in *C. elegans* model. We believe that this observation provides a new insight into *S. aureus* virulence. The possibility that a non-virulent commensal strain impacts the virulence of *S. aureus* is of great interest, considering the numbers of commensal bacteria contained in DFU (Gardner et al., 2013). Our results obtained in a model organism emphasize the importance of studying the connections between pathogenic species and the endogenous microbiota. If pathogenic bacteria are well-characterized in infection, they cannot be reduced to a single organism infecting host. All the bacteria participate to the chronicity of the wound at different levels and their virulence modulation has to be investigated to a best management of the wounds. The fact as a commensal bacterium decreases the virulence of clearly pathogenic bacteria could explain that *S. aureus* did not involve immediately an acute infection on chronic wound but rather remains in a biofilm status (which however induces a delayed healing) due to the different “environmental” conditions encountered by the pathogenic bacteria. Our results are a step in the understanding of the transition between DFU and DFI. This could also represent new ways to fight infections.

## Author contributions

JPL, CDR, OV, EL, and AS conceived and designed the experiments. CNE, OV, MFG, VC, and CD performed the experiments. MFG, AV, VC, AL provided the *Helcococcus* strains. CNE, OV, MFG, EL, AS, JPL, and CDR analyzed the data. CNE, JPL, and CDR wrote the paper. OV, MFG, AV, VC, AL, EL, and AS reviewed and edited the manuscript.

## Funding

This work was supported by INSERM.

### Conflict of interest statement

The authors declare that the research was conducted in the absence of any commercial or financial relationships that could be construed as a potential conflict of interest.
