# Supplementary material for: Decrease of Staphylococcus aureus Virulence by Helcococcus kunzii in a Caenorhabditis elegans Model
Source: Front Cell Infect Microbiol. 2017 Mar 16;7:77. doi: 10.3389/fcimb.2017.00077 (PMC5352687; doi:10.3389/fcimb.2017.00077)
Supplement: Supplementary file 1 [file DataSheet1.docx]

**Supplementary data**

**Table S1** 50% Lethal Time of *Caenorhabditis elegans* infected with the different *S. aureus* and *H. kunzii* strains and evaluation of feeding behaviour by measuring the pathogen avoidance. The results are representative of at least five independent asssays for each group of strains. *P*: Pairwise comparison between LT50s using a log rank test. NS: not significant. LT50: 50% Lethal Time.

| **Strain** | **Characteristics of the strain (References)** | **LT50 in days (IC95% inf-sup)** | **Occupancy test after 16h (%)** | *P*  OP50 vs others | *P*  NSA739 vs others | *P*  NSA1385  vs others | *P*  Newman vs others |
| --- | --- | --- | --- | --- | --- | --- | --- |
| NSA1385 | *S. aureus*, clinical, colonizing (Sotto *et al*., 2008) | 4.7 (4.5-4.8) | 98 ±2 | <0.001 | <0.001 | - | NS |
| NSA739 | *S. aureus*, clinical, infecting (Sotto *et al*., 2008) | 2.8 (2.4-3.0) | 96 ±4 | <0.001 | - | <0.001 | <0.001 |
| Newman | *S. aureus*, reference | 4.3 (4.0-4.6) | 95 ±4 | <0.001 | <0.001 | NS | - |
| H1 | *H. kunzii*, clinical, colonizing (Vergne *et al*., 2015) | 7.2 (6.4-8.0) | 100 ±0 | NS | <0.001 | <0.001 | <0.001 |
| H2 | *H. kunzii,* clinical, colonizing (Vergne *et al*., 2015) | 7.2 (6.4-8.0) | 92 ±5 | NS | <0.001 | <0.001 | <0.001 |
| H3 | *H. kunzii*, clinical, colonizing (Vergne *et al*., 2015) | 7.0 (6.4-7.6) | 95 ±3 | NS | <0.001 | <0.001 | <0.001 |
| H4 | *H. kunzii,* clinical, colonizing (Vergne *et al*., 2015) | 7.5 (6.8-8.2) | 100 ±0 | NS | <0.001 | <0.001 | <0.001 |
| H6 | *H. kunzii,* clinical, colonizing (Vergne *et al*., 2015) | 6.1 (5.9-6.5) | 97 ±3 | NS | <0.001 | <0.001 | <0.001 |
| H7 | *H. kunzii,* clinical, colonizing (Vergne *et al*., 2015) | 5.7 (5.5-5.9) | 98 ±3 | <0.001 | <0.001 | <0.001 | <0.001 |
| H8 | *H. kunzii,* clinical, colonizing (Vergne *et al*., 2015) | 6.8 (6.4-7.2) | 100 ±0 | NS | <0.001 | <0.001 | <0.001 |
| H9 | *H. kunzii,* clinical, colonizing (Vergne *et al*., 2015) | 8.0 (7.5-8.5) | 97 ±3 | NS | <0.001 | <0.001 | <0.001 |
| H10 | *H. kunzii,* clinical, colonizing (Vergne *et al*., 2015) | 5.5 (4.6-6.4) | 96 ±4 | <0.001 | <0.001 | <0.001 | <0.001 |
| H11 | *H. kunzii,* clinical, colonizing (Vergne *et al*., 2015) | 7.5 (7.3-7.7) | 100 ±0 | NS | <0.001 | <0.001 | <0.001 |
| H12 | *H. kunzii,* clinical, colonizing (Vergne *et al*., 2015) | 6.2 (5.8-6.6) | 99 ±1 | NS | <0.001 | <0.001 | <0.001 |
| H13 | *H. kunzii,* clinical, colonizing (Vergne *et al*., 2015) | 6.3 (5.8-6.8) | 100 ±0 | NS | <0.001 | <0.001 | <0.001 |
| H14 | *H. kunzii,* clinical, colonizing (Vergne *et al*., 2015) | 6.7 (5.9-7.5) | 92 ±3 | NS | <0.001 | <0.001 | <0.001 |
| H16 | *H. kunzii,* clinical, colonizing (Vergne *et al*., 2015) | 6.5 (6.2-6.7) | 100 ±0 | NS | <0.001 | <0.001 | <0.001 |
| H17a | *H. kunzii,* clinical, colonizing (Vergne *et al*., 2015) | 5.6 (5.0-6.2) | 94 ±4 | <0.001 | <0.001 | <0.001 | <0.001 |
| H17b | *H. kunzii,* clinical, colonizing (Vergne *et al*., 2015) | 7.4 (7.2-7.6) | 100 ±0 | NS | <0.001 | <0.001 | <0.001 |
| H18 | *H. kunzii,* clinical, colonizing (Vergne *et al*., 2015) | 7.1 (6.7-7.5) | 100 ±0 | NS | <0.001 | <0.001 | <0.001 |
| H19 | *H. kunzii,* clinical, colonizing (Vergne *et al*., 2015) | 6.6 (6.1-7.1) | 100 ±0 | NS | <0.001 | <0.001 | <0.001 |
| H20 | *H. kunzii,* clinical, colonizing (Vergne *et al*., 2015) | 5.8 (5.3-6.1) | 96 ±2 | <0.001 | <0.001 | <0.001 | <0.001 |
| H21 | *H. kunzii,* clinical, colonizing (Vergne *et al*., 2015) | 6.0 (5.4-6.6) | 100 ±0 | <0.001 | <0.001 | <0.001 | <0.001 |
| H22a | *H. kunzii,* clinical, colonizing (Vergne *et al*., 2015) | 7.4 (7.0-7.9) | 91 ±3 | NS | <0.001 | <0.001 | <0.001 |
| H22b | *H. kunzii,* clinical, colonizing (Vergne *et al*., 2015) | 5.6 (5.0-6.2) | 100 ±0 | <0.001 | <0.001 | <0.001 | <0.001 |
| H23 | *H. kunzii,* clinical, colonizing (Vergne *et al*., 2015) | 6.5 (6.1-7.0) | 95 ±4 | NS | <0.001 | <0.001 | <0.001 |
| OP50 | *E. coli*, control strain | 7.1 (6.6-7.7) | 100 ±0 | - | <0.001 | <0.001 | <0.001 |

**Table S2** 50% Lethal Time of *Caenorhabditis elegans* co-infected with a virulent *S. aureus* strain (NSA739) and *H. kunzii* strains and evaluation of feeding behaviour by measuring the pathogen avoidance. The results are representative of at least four independent assays for each group of strains. *P*: Pairwise comparison between LT50s using a log rank test. NS: not significant. LT50: 50% Lethal Time. Infection of nematodes with H10 or H13 followed by transfer on ^ψ^ OP50 or * *S. aureus* 12 h after.

| **Strain** | **LT50 in days**  **(IC95% inf-sup)** | **Occupancy test after 16h (%)** | *P*  OP50 vs others | *P*  NSA739 vs others | *P*  H10 vs others | *P*  H13 vs others |
| --- | --- | --- | --- | --- | --- | --- |
| NSA739 | 2.8 (2.4-3.0) | 96 ±4 | <0.001 | - | <0.001 | <0.001 |
| H10 | 5.5 (4.6-6.4) | 96 ±4 | <0.001 | <0.001 | - | NS |
| H13 | 6.2 (5.8-6.6) | 100 ±0 | NS | <0.001 | NS | - |
| H1 + NSA739 | 5.0 (4.9-5.1) | 100 ±0 | <0.001 | <0.001 | NS | <0.001 |
| H2 + NSA739 | 4.8 (4.5-5.0) | 95 ±5 | <0.001 | <0.001 | NS | <0.001 |
| H3 + NSA739 | 4.7 (4.6-4.8) | 92 ±4 | <0.001 | <0.001 | NS | <0.001 |
| H4+ NSA739 | 5.3 (5.1-5.7) | 100 ±0 | <0.001 | <0.001 | NS | NS |
| H6+ NSA739 | 4.4 (4.3-4.6) | 100 ±0 | <0.001 | <0.001 | <0.001 | <0.001 |
| H7+ NSA739 | 4.7 (4.6-4.8) | 94 ±6 | <0.001 | <0.001 | NS | <0.001 |
| H8+ NSA739 | 4.3 (4.2-4.4) | 95 ±3 | <0.001 | <0.001 | <0.001 | <0.001 |
| H9+NSA739 | 4.8 (4.7-4.9) | 100 ±0 | <0.001 | <0.001 | NS | <0.001 |
| H10+ NSA739 | 4.1 (4.0-4.3) | 92 ±5 | <0.001 | <0.001 | <0.001 | <0.001 |
| H11+ NSA739 | 5.2 (5.1-5.4) | 90 ±5 | <0.001 | <0.001 | NS | NS |
| H12+ NSA739 | 4.4 (4.3-4.5) | 100 ±0 | <0.001 | <0.001 | <0.001 | <0.001 |
| H13+ NSA739 | 5.7 (5.3-5.9) | 100 ±0 | <0.001 | <0.001 | <0.001 | NS |
| H14+ NSA739 | 4.7 (4.6-4.8) | 97 ±3 | <0.001 | <0.001 | NS | <0.001 |
| H16+ NSA739 | 5.1 (4.9-5.5) | 98 ±3 | <0.001 | <0.001 | NS | <0.001 |
| H17a+ NSA739 | 4.5 (4.3-4.6) | 100 ±0 | <0.001 | <0.001 | NS | <0.001 |
| H17b+ NSA739 | 5.3 (5.0-5.5) | 93 ±5 | <0.001 | <0.001 | NS | NS |
| H18+ NSA739 | 5.2 (5.0-5.4) | 100 ±0 | <0.001 | <0.001 | NS | NS |
| H19+NSA739 | 4.8 (4.7-4.9) | 100 ±0 | <0.001 | <0.001 | NS | <0.001 |
| H20+ NSA739 | 4.6 (4.5-4.8) | 91 ±5 | <0.001 | <0.001 | NS | <0.001 |
| H21+ NSA739 | 4.7 (4.6-4.9) | 98 ±2 | <0.001 | <0.001 | NS | <0.001 |
| H22a+ NSA739 | 4.7 (4.6-4.8) | 94 ±4 | <0.001 | <0.001 | NS | <0.001 |
| H22b+ NSA739 | 5.0 (4.8-5.3) | 100 ±0 | <0.001 | <0.001 | NS | <0.001 |
| H23+ NSA739 | 5.1 (4.9-5.3) | 98 ±2 | <0.001 | <0.001 | NS | <0.001 |
| H10> +OP50 ^ψ^ | 6.6 (6.2-6.8) | 100 ±0 | NS | <0.001 | <0.001 | NS |
| H13> +OP50 ^ψ^ | 6.2 (5.8-6.6) | 97 ±3 | NS | <0.001 | NS | NS |
| NSA739> +OP50 ^ψ^ | 2.8 (2.4-3.0) | 96 ±4 | <0.001 | NS | <0.001 | <0.001 |
| NSA1385> +OP50 ^ψ^ | 4.4 (4.0-5.1) | 100 ±0 | <0.001 | <0.001 | <0.001 | <0.001 |
| Newman> +OP50 ^ψ^ | 4.0 (3.5-4.3) | 94 ±4 | <0.001 | <0.001 | <0.001 | <0.001 |
| H10> +NSA739 * | 2.5 (2.4-2.7) | 100 ±0 | <0.001 | NS | <0.001 | <0.001 |
| H13> +NSA739 * | 4.1 (3.7-4.4) | 100 ±0 | <0.001 | <0.001 | <0.001 | <0.001 |
| OP50 | 7.1 (6.6-7.7) | 100 ±0 | - | <0.001 | <0.001 | <0.001 |

**Table S3** 50% Lethal Time of *Caenorhabditis elegans* co-infected with *S. aureus* strains and *H. kunzii* strains and evaluation of feeding behaviour by measuring the pathogen avoidance. The results are representative of at least four independent assays for each group of strains. *P*: Pairwise comparison between LT50s using a log rank test. NS: not significant. LT50: 50% Lethal Time. Infection of nematodes with H10 or H13 followed by transfer on * *S. aureus* 12 h after.

| **Strain** | **LT50 in days**  **(IC95% inf-sup)** | **Occupancy test after 16h (%)** | *P*  OP50 vs others | *P*  NSA1385 vs others | *P*  Newman vs others | *P*  H10 vs others | *P*  H13 vs others |
| --- | --- | --- | --- | --- | --- | --- | --- |
| NSA1385 | 4.7 (4.5-4.8) | 98 ±2 | <0.001 | - | NS | NS | <0.001 |
| Newman | 4.3 (4.0-4.6) | 95 ±4 | <0.001 | NS | - | <0.001 | <0.001 |
| H10 | 5.5 (4.6-6.4) | 96 ±4 | <0.001 | NS | <0.001 | - | NS |
| H13 | 6.2 (5.8-6.6) | 100 ±0 | NS | <0.001 | <0.001 | NS | - |
| H1 + NSA1385 | 5.0 (4.8-5.3) | 96 ±4 | <0.001 | NS | ) | NS | <0.001 |
| H1 + Newman | 3.3 (3.1-3.4) | 96 ±4 | <0.001 | <0.001 | NS | <0.001 | <0.001 |
| H2 + NSA1385 | 5.3 (5.1-5.5) | 100 ±0 | <0.001 | NS | - | NS | NS |
| H2 + Newman | 5.2 (5.0-5.7) | 95 ±4 | <0.001 | NS | NS | NS | <0.001 |
| H3 + NSA1385 | 4.7 (4.5-4.8) | 100 ±0 | <0.001 | NS | - | NS | <0.001 |
| H3 + Newman | 3.8 (3.6-3.9) | 92 ±3 | <0.001 | NS | NS | <0.001 | <0.001 |
| H4+ NSA1385 | 5.9 (5.8-6.1) | 100 ±0 | <0.001 | <0.001 | - | NS | NS |
| H4 + Newman | 4.8 (4.4-5.2) | 100 ±0 | <0.001 | NS | NS | NS | <0.001 |
| H6+ NSA1385 | 5.8 (5.6-6.0) | 100 ±0 | <0.001 | <0.001 | - | NS | NS |
| H6 + Newman | 4.0 (3.8-4.2) | 91 ±4 | <0.001 | NS | NS | <0.001 | <0.001 |
| H7+ NSA1385 | 5.6 (5.3-5.8) | 100 ±0 | <0.001 | NS | - | NS | NS |
| H7 + Newman | 4.4 (4.2-4.6) | 100 ±0 | <0.001 | NS | NS | <0.001 | <0.001 |
| H8+ NSA1385 | 5.9 (5.7-6.2) | 92 ±3 | <0.001 | <0.001 | - | NS | NS |
| H8 + Newman | 3.6 (3.5-3.8) | 100 ±0 | <0.001 | <0.001 | NS | <0.001 | <0.001 |
| H9+ NSA1385 | 5.0 (4.9-5.1) | 100 ±0 | <0.001 | NS | - | NS | <0.001 |
| H9 + Newman | 6.1 (5.9-6.2) | 95 ±4 | <0.001 | <0.001 | <0.001 | NS | NS |
| H10+ NSA1385 | 4.0 (3.9-4.2) | 100 ±0 | <0.001 | NS | - | <0.001 | <0.001 |
| H10 + Newman | 3.6 (3.5-3.7) | 90 ±5 | <0.001 | <0.001 | NS | <0.001 | <0.001 |
| H11+ NSA1385 | 5.3 (5.0-5.5) | 100 ±0 | <0.001 | NS | - | NS | NS |
| H11 + Newman | 4.0 (3.9-4.2) | 91 ±4 | <0.001 | NS | NS | <0.001 | <0.001 |
| H12+ NSA1385 | 5.0 (4.9-5.3) | 97 ±3 | <0.001 | NS | - | NS | <0.001 |
| H12 + Newman | 4.2 (4.1-4.3) | 90 ±5 | <0.001 | NS | NS | <0.001 | <0.001 |
| H13+ NSA1385 | 5.8 (5.7-5.9) | 100 ±0 | <0.001 | <0.001 | - | NS | NS |
| H13 + Newman | 6.3 (6.2-6.4) | 90 ±5 | NS | <0.001 | <0.001 | NS | NS |
| H14+ NSA1385 | 5.1 (4.9-5.4) | 97 ±3 | <0.001 | NS | - | NS | <0.001 |
| H14 + Newman | 5.1 (4.9-5.2) | 95 ±4 | <0.001 | NS | NS | NS | <0.001 |
| H16+ NSA1385 | 6.3 (6.1-6.4) | 90 ±5 | NS | <0.001 | - | NS | NS |
| H16 + Newman | 4.6 (4.2-5.0) | 100 ±0 | <0.001 | NS | NS | NS | <0.001 |
| H17a+ NSA1385 | 5.2 (5.0-5.4) | 100 ±0 | <0.001 | NS | - | NS | <0.001 |
| H17a + Newman | 4.1 (3.9-4.4) | 100 ±0 | <0.001 | NS | NS | <0.001 | <0.001 |
| H17b+ NSA1385 | 5.7 (5.3-6.0) | 92 ±3 | <0.001 | <0.001 | - | NS | NS |
| H17b + Newman | 5.4 (5.2-5.6) | 98 ±2 | <0.001 | NS | <0.001 | NS | NS |
| H18+ NSA1385 | 5.7 (5.3-6.1) | 100 ±0 | <0.001 | <0.001 | - | NS | NS |
| H18 + Newman | 5.9 (5.6-6.2) | 97 ±3 | <0.001 | <0.001 | <0.001 | NS | NS |
| H19+ NSA1385 | 4.8 (4.6-5.0) | 90 ±5 | <0.001 | NS | - | NS | <0.001 |
| H19 + Newman | 3.8 (3.7-3.9) | 100 ±0 | <0.001 | NS | NS | <0.001 | <0.001 |
| H20+ NSA1385 | 5.2 (5.1-5.3) | 92 ±3 | <0.001 | NS | - | NS | <0.001 |
| H20 + Newman | 4.5 (4.3-4.6) | 94 ±6 | <0.001 | NS | NS | <0.001 | <0.001 |
| H21+ NSA1385 | 4.6 (4.5-4.8) | 100 ±0 | <0.001 | NS | - | NS | <0.001 |
| H21 + Newman | 4.7 (4.3-5.0) | 94 ±6 | <0.001 | NS | NS | NS | <0.001 |
| H22a+ NSA1385 | 5.7 (5.6-5.8) | 95 ±4 | <0.001 | <0.001 | - | NS | NS |
| H22a + Newman | 6.0 (5.9-6.1) | 92 ±3 | <0.001 | <0.001 | <0.001 | NS | NS |
| H22b+ NSA1385 | 6.1 (5.8-6.4) | 100 ±0 | NS | <0.001 | - | NS | NS |
| H22b + Newman | 5.9 (5.4-6.3) | 98 ±2 | <0.001 | <0.001 | <0.001 | NS | NS |
| H23+ NSA1385 | 6.0 (5.9-6.1) | 100 ±0 | <0.001 | <0.001 | - | NS | NS |
| H23 + Newman | 7.1 (6.8-7.4) | 92 ±3 | NS | <0.001 | <0.001 | <0.001 | NS |
| H10> + NSA1385 * | 4.9 (4.7-5.1) | 100 ±0 | <0.001 | NS | - | NS | <0.001 |
| H10> + Newman * | 4.6 (4.3-4.8) | 95 ±4 | <0.001 | NS | NS | NS | <0.001 |
| H13> + NSA1385 * | 5.2 (5.0-5.3) | 100 ±0 | <0.001 | NS | - | NS | <0.001 |
| H13> + Newman * | 5.5 (5.2-5.7) | 92 ±3 | <0.001 | NS | <0.001 | NS | NS |

**Figure S1**. Growth curves of the different strains studied in presence of Nematode Growth Medium.

**Figure S2**. PFGE of the 23 *Helcococcus kunzii* studied.


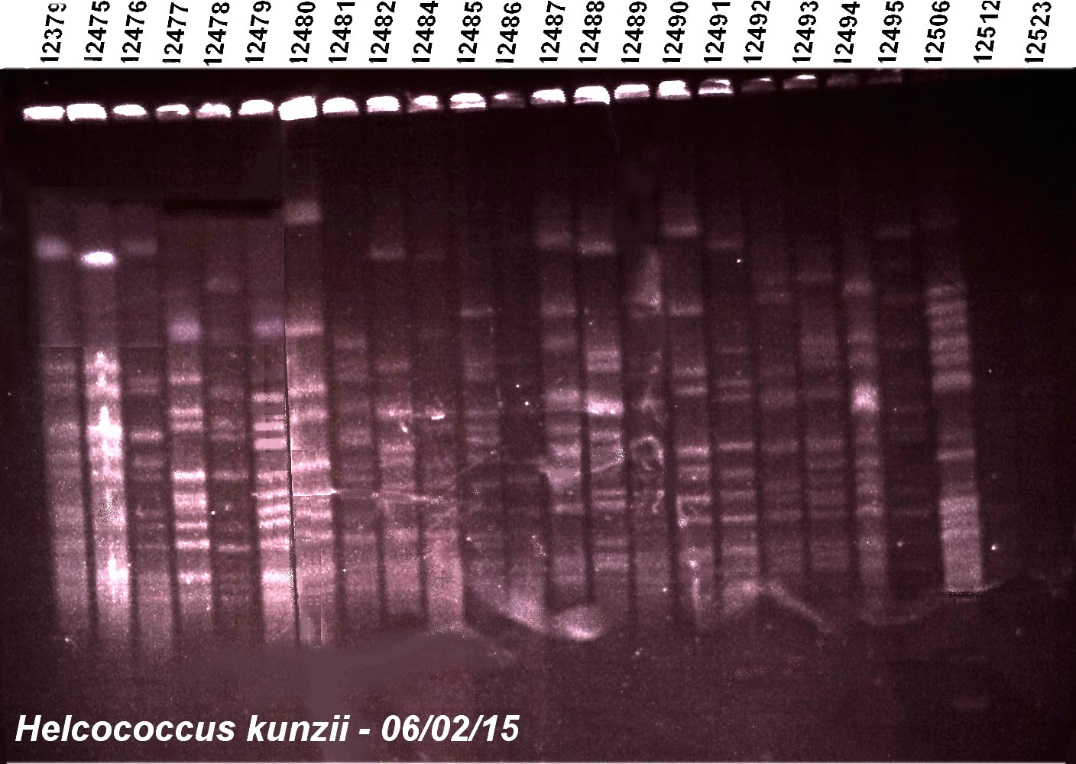


1 2 3 4 5 6 7 8 9 10 11 12 13 14 15 16 17 18 19 20 21 22 23
